# Outcome of Tendon Transfers for Radial Nerve Palsy in a Malaysian Tertiary Centre

**DOI:** 10.5704/MOJ.1803.001

**Published:** 2018-03

**Authors:** J Richford, S Abdullah, M Norhafizah, I Juliana, F Rashdeen, A Razana

**Affiliations:** Department of Orthopaedics and Traumatology, Universiti Kebangsaan Malaysia, Cheras, Malaysia; ^*^Department of Physiotherapy, Hospital Kuala Lumpur, Kuala Lumpur, Malaysia; ^**^Department of Occupational Therapy, Hospital Kuala Lumpur, Kuala Lumpur, Malaysia; ^***^Institute of Orthopaedics and Traumatology, Hospital Kuala Lumpur, Malaysia

**Keywords:** tendon transfers, high radial nerve palsy

## Abstract

Tendon transfers for radial nerve palsy is a common operation with good results. We did a retrospective study on twenty patients with radial nerve palsy who underwent tendon transfer surgery and recovered between January 2008 and December 2012. Outcomes measured were motor power of wrist extension, finger extension, grip strength and DASH scores. There was significant improvement of motor power of wrist and finger extension between the preoperative period and three months post-operatively, between the pre operative period and six months post operatively and between three and six months postoperatively (p = 0.0005). Grip strength improved significantly as well between preoperative, three and six months postoperatively (p = 0.0005). DASH scores reflecting patient satisfaction at six months postoperatively showed only mild or moderate difficulty of function.

## Introduction

Tendon transfers for radial nerve paralysis have a 100-year history with experimentation and utilization of various sets of tendons. Pronator teres is used for restoration of wrist dorsiflexion whilst flexor carpi radialis, flexor carpi ulnaris, flexor digitorum superficialis and palmaris longus are variably used in finger and thumb extension. Grip is severely impaired following loss of radial nerve function as a result of loss of wrist, metacarpophalangeal joint and thumb extension. If the radial nerve does not show any neural recovery following non-surgical or surgical repair, tendon transfer is considered the standard treatment^1^.

Factors considered in determining indication for tendon transfers in high radial nerve injury are: unlikely nerve recovery, no recovery following nerve repair surgery and failure to achieve recovery after a period of non-surgical management^2^. Outcomes of tendon transfer are favorable if there is good wrist extension, finger extension, thumb extension and hand grip^3^. Dunnet *et al* reviewed 49 cases of flexor-to-extensor tendon transfer following injury to the radial nerve with 84% improvement in hand function^4^. There is an increase of 48% in power grip and 62% in tip pinch suggesting early tendon transfer quickly restores efficient grip whilst awaiting reinnervation of wrist extensors and avoids the need for prolonged external splintage^5^.

The objective of this study was to evaluate the short-term outcome of tendon transfers in high radial nerve palsy in restoration of wrist in the Malaysian context.

## Materials and Methods

This is a retrospective study of twenty patients diagnosed with high radial nerve palsy from January 2008 until December 2012 in the Hand Unit, Institute of Orthopaedic and Traumatology of an urban government tertiary hospital. The inclusion criteria were patients with wrist and finger drop secondary to either high radial nerve injury with or without involvement of the posterior cord of the brachial plexus and those treated non-surgically for at least six months post-injury. Exclusion criteria were total brachial plexus injury (traumatic or congenital) affecting the flexor and pronator components (donor tendons) of the forearm and any musculoskeletal disorders affecting donor tendons such as cerebral palsy. We used the hand dynamometer for power grip assessment and Modified Medical Research Council (MRC) grading for muscle power assessment.

All patients underwent pronator teres (PT) to extensor carpi radialis brevis (ECRB) tendon transfer for restoration of wrist dorsiflexion, and palmaris longus (PL) to extensor pollicis longus (EPL) tendon transfer for thumb extension. However, for finger extension, fifteen underwent flexor carpi ulnaris (FCU) to extensor digitorum communis (EDC) for finger extension whilst the remaining five utilized flexor carpi ulnaris (FCU) to extensor digitorum communis (EDC). Two experienced hand surgeons working more than 10 years in the orthopaedic hand services performed the above surgeries with the first surgeon utilizing FCU and the second, FCR. For PT, a dorsal approach was used to identify the muscle at the midshaft of radius extending distally to the attachment of ECRL/B. The entire periosteum over the midshaft of radius of the PT was elevated as a single tendon unit and transferred to ECRL/B. A volar incision was used to harvest PL to EPL and FCU or FCR tendon to EDC. Both EPL and EDC were rerouted out from the 3rd and 4th dorsal compartment respectively proximal to the extensor retinaculum and inserted to their respective donor tendon. All sets of tendons were transferred subcutaneously and the Pulvertaft weave technique utilized with the tendon interwoven four times at 90 degrees to each other, using 4/0 non-absorbable sutures at each pass. During suturing, all sets of tendons were repaired and tensioned in the functional wrist position (wrist extension between 45° to 60°) whilst thumb and fingers were all in full extension.

Postoperatively, the transfers were protected with a thermoplastic splint fabricated preoperatively. Re-education of muscle-tendon unit was an important part of the rehabilitation following the tendon transfers. Patients were motivated to perform combined motions for wrist extension, pronation of forearm and simultaneous wrist extension. All patients underwent standard physiotherapy care and rehabilitation regime by one dedicated physiotherapist and one occupational therapist until six months postoperatively. An integrated team approach between the surgeon and hand therapist was conducted in this study^6^. All patients were protected by an above-elbow volar thermoplastic splint for six weeks in MCPJ, PIPJ and DIPJ full extension and wrist in 30° to 40° extension. Exercises were tailored between intermittent active flexion and passive extension according to the regime as shown in Table I. The splints were discontinued at six weeks.

**Table I:** Modified Medical Research Council (MRC) Grading for muscle power assessment with statistical value

Grade	Grade definition	Statistical value
5	Normal or hold s test position against maximal resistance	9
4+	Holds test position against moderate to strong pressure	8
4	Holds test position against moderate resistance	7
3+	Holds test position against slight resistance	6
3	Holds test position against gravity	5
2+	Moves through partial ROM against gravity	4
2	Able to move through full ROM when gravity eliminated	3
1+	Moves through partial ROM gravity eliminated	2
1	A flicker of movement is seen or felt in the muscle	1
0	No movement	0

Modified MRC was used to quantify accurately the amount of manual resistance. Adding (+) or (-) to the grades denoted the outer and inner range of motion (Table I). However, in this study there were no inner range (-) noted in both physiotherapy and surgeon medical records. In addition, usage of outer range (+) for this study was to precisely document the exact improvement. The statistical value was added for easy analysis calculation. Assessment of handgrip was done using the mean maximum handgrip of three readings in kilogram (kg) of both normal and affected hand with an interval of 5 to 10 seconds resting between each grip attempt using JAMAR handgrip dynometer^7,8^.

We utilized the Quick DASH score (based on responses to 11 questions) to assess the overall upper limb function. The DASH questionnaire was divided into 5 categories; functional activity, social activities, regular daily works, pain with numbness and sleep disturbance. DASH questions 1 to 6 were about hand function in daily life activity, question 7 was about social activities with family, friend, neighbors or groups, question 8 was regarding limitation in patient’s occupation, questions 9 and 10 were about the severity of pain and tingling (pins and needles) in patient’s arm, shoulder and hand. Question 11 was on sleep disturbances due to the affected hand. Low scores indicated no difficulty in an activity whilst high scores indicated inability to use the injured hand. The tool used a 5-point scale from which the patient could select an appropriate number corresponding to his/her severity level/ function level. The assigned values for all completed responses were simply summed up and producing lowest score of 11 (no difficulty) and highest score of 55 (unable to use the injured hand). Patients were either asked directly during the physiotherapy session, or by telephone calls at six months or more postoperatively, either by the author, medical assistant or the physiotherapist. The score scale was classified as either an excellent, good, satisfactory or poor. Table II a and b depicts the score and its interpretation^9-10^. The assigned values for all completed responses could also be transformed to a score out of 100. Quick Dash score = {[(Sum of 11 scores)/11] -1} x 25. This transformation was done to make the score easier to compare with other measures scaled based on 0-100. Score of zero indicated no disability and a score of 100 indicated complete disability^11^. Beaton *et al* have also reported that the Quick DASH score is just as reliable as the original DASH^10-12^.

**Table II: (a):** Outcome grading for Quick DASH score for total accumulation over 55 score (Accumulation score of 11 questionnaires)

Parameter	n(%)
-5	Excellent
6-15	Good
15-35	Satisfactory
>35	Poor

**Table II: (b):** Outcome grading for Quick DASH for total score transform to 100

Sum of score transform to 100 {[(Sum of 11 scores)/11] -1} x 25	Interpretation
0-29	Injury no longer considered problem
10-29	Threshold for returning to work or ready to discharge from treatment/therapy or aware of the injury but not considered problem
40-69	Moderate difficulty
70-99	Severe difficulty
100	Totally unable to utilize the hand limb

Categorical variables and baseline demographic data are described using frequencies and percentages. Continuous variables with a symmetric distribution are presented using means and standard deviations (SD) and continuous variables with a skewed distribution are presented using the median and inter-quartile range (IQR). The data was analyzed using the SPSS version 19.0 software using nonparametric test (Friedman test).

## Results

The mean age of patients was 30 years. Seventeen were male patients (85%) while three were female (15%). Malays contributed to 70% of the cases followed by Chinese (15%), Indians (10%) and other ethnicities (5%). Ninety-five percent of the study group were in employment during the time of injury. Sixty five percent involved a right-sided upper limb injury.

Comparing the muscle power in wrist and finger extensions before the surgery, three months and six months post operatively, we noted statistically significant improvements in the power of wrist extension [χ^2^ (2) = 36.521, p = 0.0005] and finger extension [χ^2^ (2) = 39.519, p = 0.0005]. The scores of muscle power were added up for each patient to produce a median value. The median for muscle power for wrist extension were 1(1-2.75), 5.5 (5 to 7) and 7.5 (7 to 9), and median for finger extension were 1 (1-1.75), 5.0 (5 to 7) and 7.5 (7 to 9) assessed preoperatively, three months and six months postoperatively. As we had assigned a numerical statistical value to the MRC grading, a value of 7.5 of both wrist and finger extensions at six months post operatively was considered equivalent to an MRC grade of 4 to 4 (+). (Table III). Our study showed significant improvements between each three-month period. We subsequently compared the muscle power between the preoperative period and 3 months, between the preoperative period and 6 months and between 3 to 6 months (Table IV). For this calculation, post-hoc analysis with Wilcoxon signed-rank tests was conducted with a Bonferroni correction. We noted significant improvements (p < 0.017) in muscle power between the preoperative period and 3 month postoperatively, between the preoperative period and 6 month postoperatively, and between 3 to 6 months postoperatively.

**Table III:** Median Variable at 50th centiles of wrist, finger extension, power of hand grip (kg) and percentage of different between injured and uninjured hand. Each variable is compared at the preoperative period, 3 and 6 months postoperative with standard deviations. Please refer to Table I for value of each score for wrist and finger extension muscle power

Variable at 50th centiles	0	Months 3	6	Chi-Square χ2	p-Value
Wrist extension power (MRC grading)	1.0 (1-2.75)	5.5 (to 7)	7.5 (to 9)	36.521	0.0005
Finger extension power (MRC grading)	1.0 (1-1.75)	5.0 (to 7)	7.5 (to 9)	39.519	0.0005
Hand grip power (kg)	8.0	15.5	32.0	40	0.0005
Percentage difference of injured hand from normal hand	74.46%	59.50%	16.88%	-	-

**Table IV:** Z-score of each variable from period between 0-3, 0-6 and 3-6 in months

Variable	Z score between each period of months		
	0-3	0-6	3-6
Wrist extension	-3.754a	-3.847a	-3.711a
Finger extension	-3.754a	-3.847a	-3.711a
Hand grip	-3.928a	-3.922a	-3.925a
p-Value of each variable	0.0005	0.0005	0.0005

Before surgery, the median for injured handgrip score was 8.0 kg as compared with 35.5 kg at the normal handgrip. There was marked mean improvement by three months postoperatively to 15.5 kg and further increase to 32.0 kg at six month postoperatively with statical value of Z = -3.928, p = 0.0005 at 3 month postoperatively Z = -3.922, p = 0.0005 at 6 month postoperatively.

The mean Quick Dash score for our patients was 23.98 +/-18.10 SD with a median (IQR) of 25.0 (Table V). This showed that overall function was satisfactory with mild to moderate difficulty at six months postoperatively. If the scores were then corrected or transformed to 100, then the mean score was 29.5 indicating good results. In this study, five of the patients had a minimum score of 11, indicating no functional difficulty in using their hand post-tendon transfer. Question number 8 scored the lowest indicating no limitation with regards to occupation or regular daily activity. Question number 2 scored the highest DASH score indicating patients were unable to do heavy household chores such as washing walls or floors.

**Table V:** Summaries of mean, median and total score of Disability of Shoulder Arm and Hand (DASH) questionnaires

Questions (DASh)	Mean Score (mean + SD)	Median Score [IQR] (at 25th and 75th centile)
DASH 1-6 Functional activity (eg open new jar, wash dishes, used knife)	11.85 + 4.61	12.00 (6.25,16.25)
DASH 7 Social activities	2.10 + 0.79	2.00 (1.25,3.00)
DASH 8 Regular daily activities	1.85 + 0.75	2.00 (1.00,2.00)
DASH 9-10 Pain and numbness	3.80 + 1.44 4.00	(2.00,4.00)
DASH 11 Disturb sleep	1.95 + 0.89	2.00 (1.00,3.00)
Total score Dash 1-11	23.98 + 18.10	25.00 (3.41,4034)

Our physiotherapy regime is outlined in Table IV. One patient defaulted physiotherapy regime due to logistical issues but assessment at six months postoperatively showed wrist extension power of at least grade 3, with marked improvement from grade 1. Her power grip was 20 kg (or 46% out of the unaffected side of 43kg). She also developed wrist and metacarpal joint stiffness resulting in inability to fully extend her wrist and fingers. At three and six months post operatively, her wrist and finger extension powers were still 3 out of 5 (MRC grading). However, she did perform her own exercises at home.

**Table VI:** Physiotherapy regime for high radial nerve transfer

Phase	Aim	Treatment
Protective phase (0 – 3/52)	To avoid tendon rupture To reduce edema To promote healing intrinsic and extrinsic wound	Full extension splint to ensure the affected wrist is in full extension with all the Metacarpophalangeal joint (MCP), fingers and thumb joint in 0 degrees Splint regime for 23 hours to allow hygiene care with full protection Review splint weekly to ensure comfort and protection of affected hand Gentle retrograde massage / compression therapy to reduce edema
Initial Active Range of movement with protection (3 – 6/52)	To promote healing of the transferred tendon To avoid joint stiff To reduce edema To minimize scar formation To promote better tendon gliding To avoid extensor lag	Changed the splint; keep the wrist in full extension with MCP joint in 70 degrees flexion and added the out rigger to encourage active range of movement of effected fingers Educate client regarding regime of new splint Start active range of movement within the splint provided Start active range of movement blocking exercise Continued scar massage
Rehabilitation phase (6 – 12/52)	To get full active range of movement of the effected hand To gain/ retrain gradually effected hand function except for strengthening To avoid formation of hypertrophic scarring	Off splint for day time and allow protective splint at night only Continue gliding exercise Continue hand function training Continue scar massage
Return to work phase (12/52 and above)	To return to previous work with good hand function	Start hand strengthening exercise Continue hand function training Start return to work

Complications such as surgical site infection, post reconstructive tendon rupture, keloid or hypertrophic scar were not encountered. There were no reports of pressure ulcers secondary to prolonged usage of protective splints.

## Discussion

Tendon injury produces considerable morbidity and disability^13^. In high and low radial nerve injury, muscle tendons innervated by Median and Ulnar nerves are used as donor tendons. Classic transfers for radial nerve palsy include those described by Brand, Jones and the modified Boyes. In this study, PT was transferred to ECRB and PL to rerouted EPL for wrist and thumb extension respectively. However, we had different methods to restore finger extension. Two experienced hand surgeons performed the operations with the first surgeon utilizing FCU and the second surgeon utilizing FCR for this purpose. The rationale for the Jones transfer (FCU to EDC) was a matter of surgeon’s preference while Brands (FCR to EDC) transfer preserves the FCU, which is an important prime ulnar stabilizer^14^. However, there was no statistical difference between both sets of tendon transfers with regards to Quick DASH score (disability of the arm, shoulder and hand), ability and time of return to job, satisfaction with the operation and range of movement^15^.

Significant improvements in wrist and finger extension and hand grip were obtained in this study. These might be due to good rehabilitation program and optimal intraoperative tensioning of the donor tendon to injured extensor tendon. Re-education of muscle-tendon unit is an important part of the rehabilitation following tendon transfers. Patients should be motivated to perform combined motions for wrist extension, pronation of the forearm and simultaneously wrist extension. Surgeon and hand therapist should implement patient integrated team approach to improve the functional outcome^12^. Any defaulted patient from physiotherapy session was contacted, and next available session was arranged. There would be at least two to three sessions per week. Moreover, protective thermoplastic slab/splint, which was templated preoperatively and were used as soon as the surgery was over, before waking up the patient from general anaesthesia. This was to prevent premature rupture of the repaired tendon while the patient was struggling during waking up after general anaesthesia.

The mean Quick-DASH score of all our patients was 23.98 +/- 18.10 SD with a median (IQR) score of 25.00. This showed that the overall patient scores were between mild to moderate difficulty at 6 months postoperatively. Interpretations of a Quick-DASH score ranging between 0-29 are where patients were “no longer considering their upper limb as problems”. For the clinician, this is the point where the patient is ready to be discharged from treatment or therapy and is ready to return to work^16^. A score ranging 40-69 indicates “having a lot of problems” (Table II a, b). In this study, there were five patients where the Quick DASH assessment was done exactly at six to eight month postoperatively. This is the reason for the above discrepancy for the Quick DASH score. In the remaining fifteen, scoring were performed more than a year after tendon transfer, reflecting a possibly better outcome. The Quick DASH score showed that pain and numbness were more tolerable postoperatively and did not disturb sleep which is an indicator of a high quality of life. Improvements of muscle power of wrist and finger extension together with handgrip are further criteria for patient satisfaction. This study affirms that surgeons would be able to reassure the patients that at six months post-operatively, they would be able to work without significant difficulties in their hand function, with no pain or numbness, as well as good social interaction.

There were some limitations in this study. Assessments after a longer interval probably of more than one year would be expected to produce even better results. Duration to return to work and duration of medical leave should have been documented together with a larger sample and a longer duration of follow-up. Clinically, range of movement should be recorded before and after surgery using goniometry measurement for more objective improvement. Due to time constraint in this study, the Quick DASH assessment was only done once at the postoperative review at six months. The evaluation should be performed before surgery, and at one or two years after surgery. We did not set an exact time for answering the questionnaire.

## Conclusion

Tendon transfers for high radial nerve palsies result in good functional outcome of wrist extension, finger extension and power of handgrip as well as good patient satisfaction through Quick DASH scores in the short term. We therefore recommend intervention if there is no recovery at three months. Early transfer of donor muscles will not compromise wrist flexion, radial or ulnar deviation or forearm pronation.

## Conflict of Interest

The authors have no conflicts of interest to disclose.
